# A review of soil waterlogging impacts, mechanisms, and adaptive strategies

**DOI:** 10.3389/fpls.2025.1545912

**Published:** 2025-02-13

**Authors:** Yusen Zhang, Xiaojuan Chen, Shiying Geng, Xiujuan Zhang

**Affiliations:** ^1^ Inner Mongolia Key Laboratory of Molecular Biology on Featured Plants, Inner Mongolia Academy of Science and Technology, Hohhot, China; ^2^ Inner Mongolia University of Bryophyte Resources and Conservation Laboratory, Inner Mongolia University, Hohhot, China

**Keywords:** waterlogging, hormone, plant, mechanism, product

## Abstract

Waterlogging is a major abiotic stress affecting plant growth and productivity. Regardless of rainfall or irrigated environments, plants frequently face waterlogging, which may range from short-term to prolonged durations. Excessive precipitation and soil moisture disrupt crop growth, not because of the water itself but due to oxygen deficiency caused by water saturation. This lack of oxygen triggers a cascade of detrimental effects. Once the soil becomes saturated, oxygen depletion leads to anaerobic respiration in plant roots, weakening their respiratory processes. Waterlogging impacts plant morphology, growth, and metabolism, often increasing ethylene production and impairing vital physiological functions. Plants respond to waterlogging stress by altering their morphological structures, energy metabolism, hormone synthesis, and signal transduction pathways. This paper synthesizes findings from previous studies to systematically analyze the effects of waterlogging on plant yield, hormone regulation, signal transduction, and adaptive responses while exploring the mechanisms underlying plant tolerance to waterlogging. For instance, waterlogging reduces crop yield and disrupts key physiological and biochemical processes, such as hormone synthesis and nutrient absorption, leading to deficiencies of essential nutrients like potassium and calcium. Under waterlogged conditions, plants exhibit morphological changes, including the formation of adventitious roots and the development of aeration tissues to enhance oxygen transport. This review also highlighted effective strategies to improve plant tolerance to waterlogging. Examples include strengthening field management practices, applying exogenous hormones such as 6-benzylaminopurine (6-BA) and γ-aminobutyric acid (GABA), overexpressing specific genes (e.g., *ZmEREB180*, *HvERF2.11*, and *RAP2.6L*), and modifying root architecture. Lastly, we discuss future challenges and propose directions for advancing research in this field.

## Introduction

Water is the source of life and a vital growth factor for plants, but it can also become a source of stress in the form of waterlogging. Excess water accumulation due to waterlogging or flooding severely disrupts normal physiological processes in plants (Irfan et al., 2010; Akhtar and Nazir, 2013). Waterlogging is commonly observed in low-lying or enclosed terrains where prolonged rainfall or storms lead to surface water accumulation. In these areas, insufficient drainage results in prolonged soil saturation.

During waterlogging stress, plant stomata close, chlorophyll degrades, and leaf light capture diminishes, ultimately reducing photosynthetic rates (Yu et al., 2019; Pan et al., 2021; Wu et al., 2022). Additionally, oxygen content in the soil decreases under hypoxic conditions, depriving plant roots of oxygen. This forces roots to shift to anaerobic respiration, impairing cellular gas exchange. Prolonged hypoxic stress exacerbates the accumulation of reactive oxygen species (ROS) and other toxic compounds, causing oxidative damage, inhibiting root respiration, and disrupting nutrient storage. These effects severely impair plant growth and development, leading to reduced crop yields or, in extreme cases, complete crop failure. When waterlogging exceeds a crop’s tolerance threshold, it can result in catastrophic reductions in agricultural productivity (Wang et al., 2023).

Flood disasters not only threaten food security in developing regions but, under the influence of global climate change, are expected to become more frequent and intense, increasing the prevalence and impact of waterlogging events (Douglas, 2009; Tong et al., 2021; Zhang et al., 2021). Consequently, understanding plant tolerance to waterlogging and the mechanisms underlying it is crucial for ensuring agricultural stability and adapting to climate change.

Waterlogging-tolerant plants exhibit several adaptive changes, with the formation of specialized root structures—such as adventitious roots, aerenchyma, and lenticels—being among the most critical. These structures help mitigate the adverse effects of waterlogging by facilitating oxygen transport. For example, under hypoxic conditions, plant roots develop aerenchyma through a process regulated by plant hormones like ethylene and auxins. Waterlogged plants also undergo significant changes in endogenous hormone levels, sucrose allocation, and carbohydrate metabolism. Ethylene plays a pivotal role in adaptive responses by promoting adventitious root development and aerenchyma formation (Huang, 2000; Irfan et al., 2010; Singh et al., 2018). In maize, for instance, ethylene signaling is essential for initiating programmed cell death (PCD) under hypoxic conditions. Additionally, flooding triggers shifts in plant gene expression that enable plants to better cope with environmental stress (Trobacher, 2009; Ebeed and El-Helely, 2021).

Exogenous hormones, including 6-benzylaminopurine (6-BA), ethylene, and abscisic acid (ABA), have been shown to mitigate the negative effects of waterlogging. These hormones not only regulate plant growth and development but also mediate stress responses. ABA, for instance, plays a central role in water stress responses, while ethylene is associated with adaptation and injury repair (Salazar et al., 2015; Chen et al., 2024). The interplay and balance between these hormones are crucial for plant survival under waterlogged conditions.

This review aims to provide a comprehensive analysis of the impact of waterlogging on plants and to explore measures to mitigate its effects. It examines advancements in research on plant tolerance to waterlogging from multiple perspectives, including morphological and anatomical adaptations, photosynthesis, respiration, ROS damage, hormone synthesis, signaling pathways, and genetic engineering approaches. Particular attention is given to how plants perceive and respond to waterlogging stress at the molecular level and how these responses are translated into morphological and physiological adaptations. Finally, this review identifies knowledge gaps in current research and proposes future directions to address these challenges. The ultimate goal is to provide a scientific foundation for breeding waterlogging-tolerant crops, enhancing agricultural sustainability and resilience in the face of climate change.

## Effects of waterlogging on plants

### Waterlogging reduces crop yields

**Figure 1:**
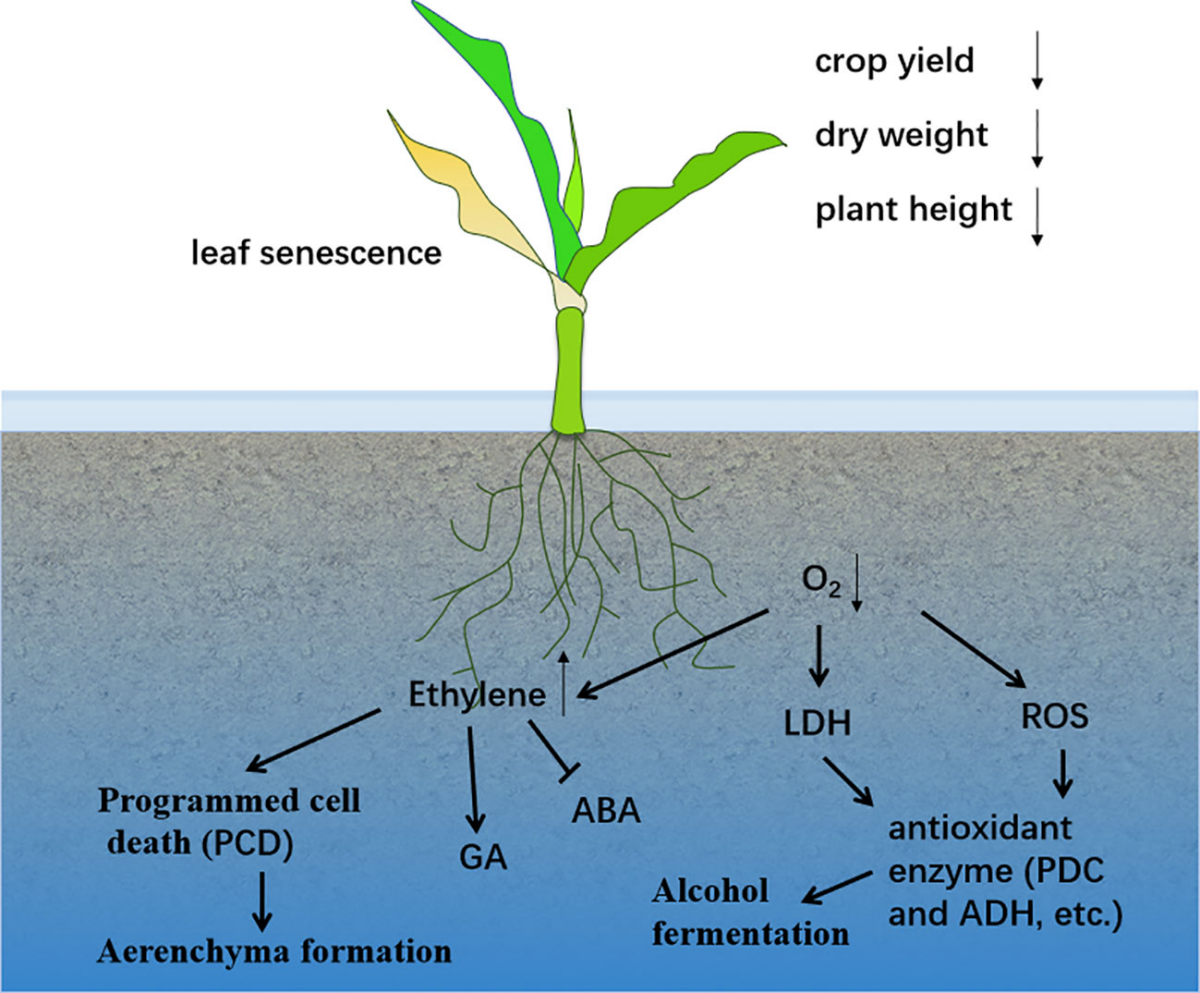
Effects of waterlogging stress on plants.

Waterlogging occurs when the water content exceeds the optimal level required for plant growth (Abiko et al., 2012). It is a major factor in reducing crop yields worldwide (Shaw and Meyer, 2015) (**Figure 1**). Efforts to mitigate waterlogging have led to substantial increases in crop yields over large areas (Bakker et al., 2010). In maize-growing regions within the subtropics, waterlogging has severely impacted production (Arora et al., 2017). Prolonged flooding reduces photosynthesis and transpiration rates, leading to a decrease in total dry weight and, ultimately, a significant reduction in spring maize yield (Tian et al., 2019). Under flooded conditions, the maximum grain filling rate decreases, the accumulation of dry matter is reduced, and the grain distribution ratio declines. Maize is particularly sensitive to waterlogging at the three-leaf stage (Ren et al., 2014). However, there are differences in the definitions and measurement methods of flood duration across various studies, which may affect the accurate assessment of flood impacts. For example, the study by Ren et al. showed that maize yield was significantly reduced after 3 days of waterlogging (Ren et al., 2014). The study by Huang et al. showed that maize yield decreased significantly after 10 days of waterlogging (Huang et al., 2022). **Table 1** provides a summary of the effects of waterlogging on various plant traits across different crop systems. 

**Table 1:** Effects of waterlogging stress (WS) on morphological and biochemical traits in different cropping systems.

Species	Affected traits	References
*Triticum* *aestivum* L.	Dry weight of stem and root ↓ Length of root ↓ Root-to-shoot ratio ↓ Root aerenchyma ↑	(Haque et al., 2011)
*Zea mays* L. (DH605 and ZD958)	Height of plant and ear ↓ Leaf area index ↓ Yield ↓ Bald tip ↑	(Ren et al., 2014)
Soybean	Number of pods↓ Dry weight of shoots and roots↓ Number of nodules↓ Adventitious roots ↑ Aerenchyma ↑	(Ploschuk et al., 2022)
*Vigna unguiculata* (L.) Walp.	Number of nodes↓ Leaf area↓ Stomata↑	(Olorunwa et al., 2022)
*Brassica napus* L.	Branch height↑ Number of branches↓ Branch height↓ Main root length↓	(Xu et al., 2015)
*Gossypium hirsutum* L.	Cotton boll weight↓ Adventitious roots↑ Aerenchyma↑	(Zhang et al., 2021)

(↑indicate up-regulate, ↓indicate down-regulate).

Waterlogging has been shown to significantly reduce wheat yields (Yaduvanshi et al., 2012; Ploschuk et al., 2018). The impact of waterlogging on crop performance depends on the species’ sensitivity and the developmental stage at which waterlogging occurs. In wheat, the period from the seventh leaf of the main stem to the flowering stage is particularly critical, as waterlogging during this time causes the greatest yield losses (de San Celedonio et al., 2014). Stem elongation is especially vulnerable to waterlogging, with grain yield declining as the duration of waterlogging increases (Ding et al., 2020). Waterlogging during the tillering stage also significantly reduces grain yield (Musgrave, 1994; Wu et al., 2015), with the most substantial yield losses occurring under waterlogging at the third leaf stage. Notably, when waterlogging treatments last beyond 6 days at any growth stage, yield reductions become particularly pronounced (Koyama et al., 2019). Compared with control conditions, waterlogging at the four-leaf stage leads to significant decreases in plant biomass, panicle number, and overall yield (Farkas et al., 2020; Li et al., 2024). Although an increase in panicle number and grain weight may partially offset the losses, early waterlogging generally results in net yield reductions (Tian et al., 2019; Ploschuk et al., 2020). In cotton, yield losses are closely tied to reduced boll numbers caused by waterlogging, with early waterlogging having a more severe impact than late flooding (Bange et al., 2004; Zhang et al., 2016b).

Similarly, waterlogging stress adversely affects rapeseed growth and development, resulting in yield reductions. The tolerance of rapeseed to waterlogging varies across growth stages (Zou et al., 2014; Liu et al., 2020a). Traits such as branch height and thousand-seed weight are less affected, but other growth and yield components are significantly impacted (Xu et al., 2015). Waterlogging during the stem elongation stage disrupts growth and nutrient accumulation, ultimately leading to yield declines (Wollmer et al., 2018).

### Plant root programmed cell death and aerenchyma formation under waterlogging

Plants exhibit stress-specific adaptive responses along with mechanisms that protect against multiple environmental stresses (Chinnusamy et al., 2004). Under waterlogged conditions, plants undergo various morphological changes to adapt, including the formation of adventitious roots, initiation of cell hypertrophy, development of aerated tissues (aerenchyma), establishment of radial oxygen loss (ROL) barriers, rapid expansion of apical meristems, and the formation of air films on the upper cuticle (Jackson, 2002; Bailey-Serres et al., 2012; Yohan et al., 2017; Liu et al., 2020b).

Aerenchyma tissues, which extend vertically and continuously from shoots to roots, are formed through the division or degradation of specific cells. These structures are a critical adaptive strategy for coping with waterlogging stress. The mechanism of aerenchyma formation begins under hypoxic conditions, where root and microbial activity consume oxygen, leading to an anaerobic environment that promotes ethylene production. Elevated ethylene levels activate enzymes such as cellulase that facilitate cell division or cortical cell collapse, resulting in aerenchyma formation (Yin et al., 2013). At the same time, hypoxic environments can induce the expression of cellulose-degrading enzymes in cortical cells. Hypoxic environments also induce the expression of cellulase in cortical cells, which increases the porosity of root tissues and optimizes oxygen diffusion from shoots to root tips through longitudinal aerenchyma channels (Pedersen et al., 2021; Shiono et al., 2008; Yamauchi et al., 2013).

Rice (*Oryza sativa* L.) demonstrates unique adaptations to waterlogging compared to other cereal crops. It thrives in paddy fields due to its high tolerance to waterlogging, forming soluble aerenchyma tissues and ROL barriers in roots to supply oxygen to the root tips (Nishiuchi et al., 2012). Waterlogging reduces the development of lateral roots along the taproot axis (Cardoso et al., 2014), while increased porosity of adventitious roots, associated with aerenchyma formation, supports root cap biomass under deoxygenated conditions (Thomas et al., 2005; Broughton et al., 2015). However, there are some contradictions between different studies regarding the specific mechanisms of aerenchyma formation. For instance, some studies have emphasized the crucial role of ethylene in programmed cell death (Jackson and Armstrong, 1999; Drew et al., 2000; Subbaiah and Sachs, 2003; Yamauchi et al., 2013), while others suggest that other hormones such as gibberellins and auxins may also be involved (Konings and de Wolf, 1984; Ho et al., 2015; Shimamura et al., 2016; Rajhi and Mhadhbi, 2019; Yamauchi et al., 2019). This contradiction may arise from differences in the plant species and experimental conditions used in various studies, and further systematic research is needed to resolve it. Plant height, tillering yield, leaf area, and total biomass are negatively affected by waterlogging stress during early growth stages (Gomathi et al., 2015) (**Figure 1**). Cortical cell death during aerenchyma formation is a programmed cell death process induced by waterlogging (Jiang et al., 2010). In many crops, aerenchyma formation occurs from gas spaces created by the death and collapse of cortical cells (Zhang et al., 2015). Over time, aerenchyma increases with prolonged waterlogging, but excessive waterlogging leads to tissue decay, root length reduction, and aeration tissue degradation (Marashi and Mojaddam, 2014). Sucrose is required for the formation of aerated cork in soybean hypocotyls (Takahashi et al., 2018). The induction of aerenchyma is influenced by genetics and tissue-specific regulatory processes (Muhlenbock et al., 2007; Herzog et al., 2016). It is associated with the activity of cell wall loosening and antioxidant enzymes (de Souza et al., 2017). Enhanced aerenchyma formation in adventitious roots has been linked to increased tolerance to waterlogging in barley (Zhang et al., 2015; Liu et al., 2021; Yan et al., 2022; Liu et al., 2023). Recent studies have highlighted that increasing the proportion of root-aerated tissues improves tolerance to waterlogging in crops like rice and maize. Genotypes with higher aerenchyma-forming capacity exhibit greater biomass accumulation and improved performance under waterlogged conditions compared to non-waterlogged controls (Gong et al., 2019; Hu et al., 2022). These studies may overemphasize the role of aerenchyma in flood tolerance while neglecting the influence of other factors, such as the morphological structure and physiological adjustments of the root system. For example, although the formation of aerenchyma can promote oxygen diffusion, its impact on overall plant growth and yield needs to be comprehensively assessed in conjunction with other physiological indicators. It should not be simplistically regarded as the sole determinant of flood tolerance.

### Changes in hormone levels under waterlogged conditions

Plant hormones are small molecules derived from various metabolic pathways and play a crucial role in regulating plant growth and development (Cheng et al., 2013).

Ethylene, the only gaseous plant hormone, is a key growth regulator involved in processes such as leaf development, fruit ripening, and germination stimulation (Van de Poel et al., 2015; Bashar, 2018; Huang et al., 2019). Ethylene biosynthesis begins with methionine, which is converted to *S*-adenosylmethionine (SAM) by *S*-adenosylmethionine synthetase (SAMS). SAM is then converted to 1-aminocyclopropane-1-carboxylic acid (ACC), the direct precursor of ethylene, by ACC synthase (ACS). Under flooding conditions, ACC is synthesized in plant roots and transported to aerobic regions where it is converted to ethylene by ACC oxidase (ACO), another key enzyme in ethylene biosynthesis (Yang and Hoffman, 1984; Jackson and Campbell, 1975; Bradford and Yang, 1980). Waterlogging induces an increase in ACO activity, which contributes to the formation of adventitious roots.

Aerenchyma formation is another ethylene-triggered adaptation that enhances oxygen availability during waterlogging, helping plants avoid anaerobic conditions (Ahmed et al., 2013; Geng et al., 2023). Although ACC conversion to ethylene requires oxygen, this process is hindered under hypoxic conditions in waterlogged root cells. Consequently, ACC is transported from anaerobic root cells to aerobic parts of the roots or shoots, where ethylene production occurs. Aerenchyma formation is commonly observed in roots exposed to low oxygen levels (Hossain and Uddin, 2011; Ahmed et al., 2013; Yukiyoshi and Karahara, 2014; Zhang et al., 2016a). Research on the specific transport mechanisms of ACC in root systems and its functional differences among various plant species is still relatively limited, requiring further experimental validation and comparative studies.

In addition to ethylene, other plant hormones such as ABA and gibberellins (GA) also play critical roles in tolerance to waterlogging. The interactions between ethylene, ABA, and GA have been extensively studied and are crucial for adaptive responses. For instance, in swamp grasses, flooding-induced ethylene accumulation inhibits ABA synthesis and promotes its degradation, reducing ABA levels and enabling rapid petiole elongation (Benschop et al., 2005). Similarly, in deep-water rice, ethylene enhances GA-triggered elongation by promoting ABA degradation, thereby alleviating waterlogging stress (Kende et al., 1998; Kendrick and Chang, 2008). The coordination between ethylene, GA, and ABA regulates adventitious root formation and shoot elongation in flooded rice (Steffens et al., 2006). These studies have often focused on specific plant species and environmental conditions, and extensive research on the interactions of plant hormones in different ecosystems is lacking. Furthermore, the analysis of hormone signaling pathways in these studies is not sufficiently in-depth, requiring more molecular biology and genomics research to reveal their complex mechanisms.

Hypoxia in roots disturbs endogenous hormone levels, reducing water and nutrient absorption (Anfang and Shani, 2021). Studies have shown that cytokinin (CK) and GA synergistically reduce ROS accumulation in mung bean under waterlogged conditions, mitigating wet and waterlogging damage (Islam et al., 2022). In deep-water rice, waterlogging-induced stem elongation is attributed to reduced ABA levels and increased GA levels (Adak et al., 2021). The ratio of ethylene, methionine, and GA in waterlogging-tolerant lines is significantly higher than in sensitive lines (Kim et al., 2015). Ethylene generation under waterlogging inhibits root elongation, decreases ABA content, and increases GA levels, leading to adventitious root formation (Loreti et al., 2016; Vidoz et al., 2016; Rasmussen et al., 2017) (**Figure 1**). Ethylene also induces aerenchyma formation and promotes ethanol fermentation under waterlogged conditions (Yin et al., 2013). Some studies may rely too heavily on short-term experimental data, neglecting the dynamic process of hormone level changes under long-term flooding conditions. This can lead to an incomplete understanding of plant adaptation mechanisms.

### Metabolic pathways in waterlogging

Waterlogging has a strong effect on the carbon metabolism of the entire plant (Yang et al., 2024). After the occurrence of waterlogging, the oxygen content of the underwater soil decreases, and plant cells sense the hypoxia signal, making adaptive adjustments [for example, pyruvate decarboxylase (PDC) and alcohol dehydrogenase (ADH) synthesis increases to produce ethanol] (Pan et al., 2021). At the same time, the metabolic pathway of plant cells also changes, which is characterized by anaerobic fermentation instead of aerobic respiration. In beech leaves, sugar also accumulates in the ligaments during waterlogging. Ethanol is transported from the roots to the leaves, which helps to reduce the concentration of toxic ethanol in the roots and supports C metabolism at the whole plant level (Ferner et al., 2012). During the long period of waterlogging, the carbohydrate concentration in seedling roots changes, and the ability to provide substrate for alcohol fermentation differs. Another difference between seedlings is associated with the γ-aminobutyric acid (GABA) shunt, which leads to the accumulation of alanine in waterlogged trees, suggesting that differences in the conversion from GABA to alanine may lead to the accumulation of phytotoxic levels of intermediates (Jaeger et al., 2009). Waterlogging has a greater impact on the energy and carbon metabolism of trees, which consume more carbohydrates per unit of time to produce enough energy to compensate for the low energy produced by fermentation (Kreuzwieser et al., 2004). Such adjustments in energy metabolism may not always be effective. For example (Bailey-Serres and Voesenek, 2008; Phukan et al., 2016), under extreme flooding conditions, plants may not be able to fully compensate for the energy deficit by increasing the consumption of carbohydrates, which can lead to growth stagnation or even death.

Sucrose synthase (SuSy) is significantly induced by waterlogging and is the main enzyme that causes sucrose degradation (Kuai et al., 2016). The effects of different waterlogging times on leaf carbohydrate metabolism observed by the authors of the above study were observed to be quite different. Net photosynthetic rate and carbohydrate metabolism were most affected by 6 days of submersion and high temperature, and SuSy activity was enhanced. Early transient waterlogging stress not only promoted leaf photosynthesis but also inhibited sucrose degradation, thus improving sucrose composition and accumulation (Wang et al., 2017). The induction of SuSy activity was the most significant, indicating that it played a key role in sucrose metabolism after submersion (Kuai et al., 2014). The enhancement of SuSy activity is associated with the regulation of various signaling molecules, but the specific roles of these signaling molecules and their differences among different plant species have not been fully investigated.

### Genetic changes under waterlogged conditions

Plants respond to hypoxia caused by waterlogging by altering transcription and translation, affecting signal transduction, carbon metabolism, and amino acid metabolism (Zou et al., 2010). Oxygen deficiency disrupts ion balance in plant cells, increasing energy demand. Genes encoding anaerobic proteins (ANPs) are rapidly activated under mild hypoxia and quickly deactivated during reoxygenation, indicating a precise oxygen-sensing mechanism in plant cells. Transient changes in Ca^2+^ and H^+^ are detected and amplified, leading to prolonged biochemical and physiological responses (Lokdarshi et al., 2016). *CML38*, a calcium sensor protein, plays a central role in hypoxia responses and accumulates in stress granules and messenger RNA ribonucleoprotein (mRNP) complexes (Lee et al., 2014). Although the role of *CML38* in hypoxia response has been recognized, its specific functions under different flooding conditions and in different plant species, along with its interactions with other signaling molecules, still require further experimental validation.

Waterlogging reduces chlorophyll content and accelerates senescence in rapeseed seedlings, which is linked to significant changes in gene expression in aerial leaves (Kreuzwieser and Rennenberg, 2014). Trees initially shift from mitochondrial respiration to lactic acid fermentation during hypoxia, which is marked by metabolite and lactate dehydrogenase (LDH) gene expression changes. Lactic acid accumulation acidifies the cytoplasmic pH, reducing LDH activity and promoting alcoholic fermentation. This process involves increased PDC and ADH activities in flooded roots, resulting in the production of ethanol (Kreuzwieser et al., 2009). Elevated transcript levels of PDC and ADH have been observed in several tree species, including poplar, within 1 hour of oxygen depletion (Sairam et al., 2009). However, there is still a lack of research on the specific mechanisms of this metabolic shift and its impact on long-term plant growth. For example, the role of lactic acid fermentation may vary among different plant species, and under long-term flooding conditions, plants may not be able to rely solely on this metabolic pathway to maintain normal physiological functions.

In mung bean, the sucrose synthase (SS) and ADH genes provide mechanisms for waterlogging tolerance by supporting glycolysis and NADH recycling through ADH activity (Zhang et al., 2017b). Cotton leaves under waterlogged conditions showed differential expression of 794 upregulated and 1,018 downregulated genes, with Kyoto Encyclopedia of Genes and Genomes (KEGG) analysis highlighting changes in flavonoid biosynthesis, oxidative phosphorylation, and anaerobic fermentation-related genes. The genetic genes related to ethylene signaling, cell wall growth and modification, hormone response, starch metabolism and nitrogen metabolism were significantly upregulated (Christianson et al., 2010). Within 4 hours of waterlogging, 1,012 genes in cotton leaves were altered, many of which were involved in glycolysis, fermentation, and mitochondrial electron transfer (Xu et al., 2017). The specific biological significance of the different gene expression changes and their dynamic regulation under varying degrees of flooding still need to be investigated.

Adventitious root primordium activation in cucumber is linked to hormone signal transduction and carbohydrate pathways, with ethylene playing a key role (Haque et al., 2014). In wheat, the *Tabwpr-1.2* gene may regulate root cortical tissue changes through ACC or H_2_O_2_ signaling (Zhao et al., 2018). Flooded chrysanthemum cultivars exhibited distinct transcription factor responses linked to hormone pathways (Su et al., 2019). Genome-wide association study (GWAS) analysis has identified candidate genes for complex waterlogging traits based on single-nucleotide polymorphism markers (Zhang et al., 2017a). However, further research is needed to validate the functions of these candidate genes under different environmental conditions and to explore their applications.

Under submersion, genes encoding ADH and PDC are transiently expressed in many species, with prolonged activation in *Dendranthema zawadskii* roots (Yin et al., 2013). In kiwifruit, ADH genes in roots are significantly upregulated after waterlogging (Nguyen et al., 2018). Inhibition of root tip elongation under hypoxia correlates with upregulated genes for auxin (*YUC1* and *PIN9*), jasmonic acid (*AOC1* and *JAR1*), and GA metabolism (Oskuei et al., 2017).

Continuous waterlogging affects photosynthesis, with reduced activity of photosynthesis-related enzymes like hydroxypyruvate reductase in soybean leaves (Jung et al., 2019). Under waterlogging stress, sesame leaves upregulate proteins such as ATP synthase, heat shock protein, glutamine synthase, and superoxide dismutase (Le Provost et al., 2012). In contrast, sessile and pedunculate oaks showed no significant changes in key fermentation and oxidative stress-related genes under waterlogged conditions (Zhai et al., 2013). However, the regulatory mechanisms of these genes in different plant species and their specific roles in flood adaptation still require to be elucidated by further molecular biological research.

**Figure 2:**
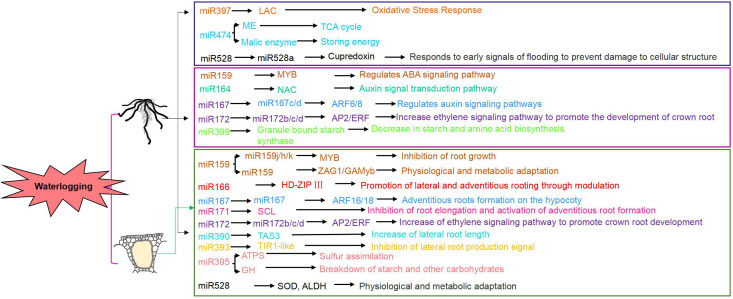
Changes in miRNA and target genes under waterlogging stress.

MicroRNAs (miRNAs) are key regulators in plant responses to abiotic stress. MiR167 and miR172 regulate crown root development under waterlogging (Liu et al., 2012). In maize, miR159, miR164, miR393, miR408, and miR528 modulate post-transcriptional mechanisms in different inbred lines under short-term waterlogging (Zhang et al., 2008) (**Figure 2**). Early induction of zma-miR166, zma-miR167, and zma-miR171 targets transcription factors like HD-ZIP and auxin response factors. Other miRNAs, including zma-miR159 and osa-miR528-like, regulate carbohydrate and energy metabolism pathways involving starch synthetase and ATPase (Zaidi et al., 2015).

Zaidi et al. identified five quantitative trait loci (QTLs) that collectively accounted for approximately 30% of the phenotypic variation in a family of flooded recombinant inbred lines (RILs). Additionally, they identified 13 QTLs associated with secondary traits related to waterlogging tolerance, with each QTL explaining between 3% and 14% of the phenotypic variation (Zaidi et al., 2015). Among these, *GRMZM2G055704* has been identified as a candidate gene responsive to waterlogging (Du et al., 2017). However, there is still insufficient research on the specific roles of miRNAs under waterlogging stress and their interactions with other regulatory factors. For example, the regulatory mechanisms of miRNAs may vary depending on the plant species and growth stage, and currently, there is limited understanding of the dynamic regulatory processes of miRNAs under different levels of waterlogging.

## Management methods to relieve waterlogging stress

### Drainage and fertilization alleviate the effects of waterlogging on plants

Drainage is an effective measure to alleviate the effects of waterlogging on plants. A proper drainage system facilitates the rapid removal of excess water from the soil, improving soil aeration and providing sufficient oxygen to plant roots, thereby reducing the risk of root suffocation. Effective drainage enhances soil permeability, preventing water accumulation in the root zone and alleviating waterlogging stress on crops. In poorly drained soils, even a daily rainfall of just 3 cm to 5 cm can significantly hinder crop growth (Linkemer et al., 1998). This illustrates how drainage can mitigate the harmful effects of waterlogging. In the Midwestern United States, the implementation of groundwater level control structures with free drainage has been shown to improve crop yields and reduce fertilizer loss. Studies have indicated that tile drainage structures enhance soil moisture conditions, allowing soybean planting to occur 17 days earlier than on undrained plots, resulting in yield increases of 9%–22% (Nelson et al., 2011).

Plants require various nutrients for growth, including nitrogen (N), phosphorus (P), potassium (K), calcium (Ca), and magnesium (Mg). Nitrogen significantly impacts plant growth; however, waterlogging triggers a series of redox reactions in the soil that produce harmful substances, such as high concentrations of inorganic ions (e.g., Mn^2+^ and Fe^2+^) and low-molecular weight organic compounds (e.g., ethanol, acetic acid, propionic acid, and butyric acid). These free acids strongly inhibit root metabolic processes and growth. Waterlogging also severely impedes the mineralization of organic matter, leading to reduced nitrogen release and nutrient deficiencies in plants. The increased activity of denitrifying bacteria can further exacerbate nitrogen loss, resulting in symptoms of nitrogen deficiency, such as leaf chlorosis, shortly after waterlogging occurs. Applying nitrogen fertilizer to the soil can help mitigate or eliminate these deficiencies (**Table 2**). For instance, lulo (*Solanum quitoense*), a species highly susceptible to waterlogging, benefits from foliar nitrogen fertilization, which helps alleviate the negative effects of abiotic stress (Flórez-Velasco et al., 2015; Jiménez et al., 2015). Additionally, the use of nitrification inhibitors in waterlogged soils has been shown to improve corn yield and nitrogen use efficiency (Ren et al., 2017a).

**Table 2:** Strategies to relieve waterlogging stress.

Species	Strategies to alleviate waterlogging stress	Key outcomes
Lulo	Nitrogen spraying	Increase in photochemical efficiency and chlorophyll concentration
Maize	Exogenous application of 6-BA	Increase in plant height and grain yield
	Exogenous application of GABA	Enhance in shoot and root dry matter
	Application of GA3	Increase in leaf area and reduction in stomatal diffusion resistance
	Application of nitrapyrin	Increase in grain yield and nitrogen utilization
	MiRNA	Transcriptional regulation and ROS elimination
	*GRMZM2G012046*, *GRMZM2G009808*, *GRMZM2G137108*, *GRMZM2G369629* (*AGPV1*)	Improvement of dry matter and plant height
	*ZmEREB180*	Promotion of formation of adventitious roots and regulation of antioxidant levels
	6-BA	Improvement of grain weight and volume and fatty acid metabolism
*Arabidopsis*	*HvERF2.11*	Improvement of antioxidant enzymes and ADH enzyme activities
	*RAP2.6L*	Delay of waterlogging-induced premature senility
Cotton	Application of AVG	Inhibition of ethylene synthesis and maintenance of photosynthetic capacity
Pepper	Application of MeJA	Modulation of antioxidant enzyme activity and root respiratory metabolism
Cucumber	Nitric oxide spraying	Enhancement of the antioxidant enzyme activity
Rapeseed	Pretreatment with uniconazole	Increase in leaf area and number of green leaves number
Wheat	Application of hardener	Improvement of dry matter and grain yield
	*TaERFVII.1*	Increase in grain weight per plant and chlorophyll content of leaf
Tobacco	Pretreatment with riboflavin	Delay of leaf senescence and extension of survival time
Soybean	ETP	Inducement of adventitious root initiation and increase of root surface area
	*qWT_Gm03*	More effective water/nutrient absorption
Pedunculate oak	*LRR* gene	Overexpression of the ADH gene
*Chrysanthemum zawadskii*	*CgACO*	Accumulation of ethylene

6-BA, 6-benzylaminopurine; GABA, γ-aminobutyric acid; GA3, gibberellic acid; ROS, reactive oxygen species; ADH, alcohol dehydrogenase; AVG, aminoethoxyvinylglycine; MeJA, methyl jasmonate; ETP, ethephon.

Phosphorus plays a crucial role in early plant growth, as plants rely on internally stored phosphorus to meet the nutritional demands of various parts, with the root system requiring more phosphorus than the stems and leaves (Khan et al., 2023). Under waterlogging stress, reduced soil oxygen levels can affect the chemical properties of metal cations like Fe³^+^, which in turn influences phosphorus fixation, decreasing its availability, solubility, and transport (Jayarathne et al., 2016).

Potassium is essential for plant development, growth, and productivity, playing a key role in cellular structure and physiological functions (Pettigrew, 2008). Adequate potassium application helps protect soybean crops from nutrient deficiencies and aids in recovery from waterlogging damage. It enhances stress tolerance and increases yield under waterlogged conditions by boosting the activity of antioxidant enzymes such as catalase (CAT) and superoxide dismutase (SOD). This reduces the production of reactive oxygen species (H_2_O_2_ and O_2_^−^), lowers malondialdehyde (MDA) content in cotton leaves, and improves plant resistance to waterlogging (Wang et al., 2022).

Calcium is a critical nutrient needed during all stages of plant growth. It enhances plant tolerance to abiotic stress by regulating cellular osmotic pressure, increasing the solubility of substances or inorganic ions, stabilizing calcium-ATPase activity, and promoting stomatal closure under ABA induction (Mohanta et al., 2018). Exogenous calcium (Ca^2+^) has been shown to improve the ability of pepper plants to withstand waterlogging stress, maintaining strong nutrient absorption capacity and proper functioning of the nutrient transport system (Ou et al., 2017).

Magnesium is essential for photosynthesis, enzyme activation, protein synthesis, and root development, playing a vital role in plant growth and stress tolerance (Tian et al., 2021). Waterlogging can lead to significant magnesium losses in the soil, and magnesium deficiency in plants can result in the accumulation of sucrose in chloroplasts, reducing photosynthetic efficiency and altering chlorophyll composition. This often manifests as leaf yellowing or necrosis (Wang et al., 2020). Pretreatment with 5 g of magnesium peroxide (MgO) in potted papaya plants can increase total dry weight and leaf area, promoting plant growth under waterlogging stress (Thani et al., 2016).

### Hormone alleviates the effects of waterlogging on plants

Exogenous application of 6-BA can effectively mitigate the adverse effects of flooding on carbon metabolism, improve the leaf area index, and alleviate waterlogging’s detrimental impacts on ultrastructure. This treatment can reduce the destruction of oxidase activity, lower abscisic acid levels, and enhance the contents of indole-3-acetic acid, zeatin nucleoside, and GA during the filling period. Consequently, this leads to decreased MDA content, increased chlorophyll levels, delayed leaf senescence, improved photosynthetic performance, and enhanced grain filling rate and yield in summer maize (Ren et al., 2016, 2017b, 2018, 2019).

The application of exogenous GABA significantly increases both aboveground and root dry matter weight, enhances photosynthetic rates and chlorophyll content, and boosts the activities of antioxidant enzymes such as SOD, peroxidase (POD), CAT, and glutamate reductase (GR), while reducing MDA content. GABA activates the antioxidant defense system by downregulating ROS production, improves chloroplast ultrastructure and photosynthetic characteristics, and promotes the growth of maize seedlings under waterlogging stress (Salah et al., 2019).

Aminoethoxyvinylglycine (AVG) inhibits ethylene accumulation in leaves, thereby improving growth, nitrogen absorption, and photosynthetic parameters. This inhibition of ethylene by AVG may help limit yield loss in flooded cotton (Najeeb et al., 2015). Gibberellic acid (GA3) application increases leaf area and the net photosynthetic rate while significantly reducing stomatal diffusion resistance on both leaf surfaces. However, it does not affect leaf water potential or chlorophyll content. Thus, GA3 reduces the impact of waterlogging on leaf area, stomatal diffusion resistance, and net photosynthetic rate (Goswami and Dayal, 1987; Leul and Zhou, 1998).

Methyl jasmonate (MeJA) treatment increases the activity of antioxidant enzymes, proline, soluble sugar contents, and ADH activity while reducing relative conductivity, MDA levels, and hydroxyl radical (–OH) accumulation. It also maintains higher activities of root malate dehydrogenase (MDH) and succinate dehydrogenase (SDH), promoting aerobic respiration metabolism. Overall, MeJA reduces flood damage to pepper plants by modulating osmotic substance content, antioxidant enzyme activity, and root respiration metabolism, and spraying has a beneficial effect prior to waterlogging (Ouli-Jun et al., 2017).

Spraying sodium nitroprusside (SNP) as a source of nitric oxide (NO) on cucumber seedlings significantly alleviates waterlogging stress, leading to increased plant height, fresh weight, and dry weight under waterlogged conditions. SNP treatment enhances antioxidant enzyme activity throughout the waterlogging period, maintains chlorophyll and protein contents, and reduces MDA levels. Therefore, NO protects plants from oxidative damage and promotes growth by activating antioxidant enzymes in the leaves, thereby alleviating membrane damage (Fan et al., 2014).

Pretreatment of rapeseed seedlings significantly enhances seedling height, the number of green leaves, and leaf area per plant after waterlogging, resulting in increased root and total dry weight (Sairam et al., 2009). Applying a hardener to waterlogged plants before flowering effectively improves the tolerance of wheat to waterlogging during the reproductive period (Li et al., 2011). Riboflavin can delay leaf senescence and prolong survival time, indicating its potential to improve plant tolerance to waterlogging. In tobacco, riboflavin pretreatment enhances stomatal closure and decreases chlorophyll content and antioxidant enzyme activity while significantly increasing lipid peroxidation, H_2_O_2_ accumulation, and total ascorbic acid and glutathione levels (Deng and Jin, 2013).

Ethephon (ETP) significantly increases photosynthetic pigment content and endogenous gas levels and alleviates waterlogging stress. ETP treatment activates amino acid content under waterlogging stress. It can induce outer membrane formation, increase root surface area, and significantly enhance the expression of glutathione transferase and the relative activity of glutathione. ETP also upregulates soybean protein content and glutathione *S*-transferase DHAR2 under waterlogged conditions, promoting plant growth and reducing waterlogging stress (Kim et al., 2018a, 2018b).

ACC deaminase strains protect plants by increasing ethylene production, thereby reducing the negative impact of waterlogging on plant yield (Barnawal et al., 2012).

### The genetic changes alleviated waterlogging stress

During waterlogging, plants undergo three primary fermentation pathways: lactic acid, ethanol, and plant-specific pathways. Research has shown that the application of 6-BA significantly regulates protein metabolism, ROS scavenging, and fatty acid metabolism-related proteins. This suggests that 6-BA enhances the defense response in summer maize against waterlogging by improving key proteins related to ROS scavenging and fatty acid metabolism, ultimately boosting drought resistance.

Plants respond to waterlogging stress through a complex alteration in gene expression that is tightly regulated at multiple levels, from epigenetic modifications to transcription and translation. Different plant species exhibit unique responses to waterlogging stress, which are generally classified into two opposing strategies: “quiescence” and “escape”. These responses are mainly mediated by ethylene (ETH)-induced transcription factors such as SNORKEL (*SK*) and SUBMERGENCE1 (*Sub1*).

#### Quiescence response

The *Sub1A* transcription factor inhibits ETH production and the expression of downstream ETH-related genes, promoting brassinosteroid (BR) synthesis and activating GA biosynthesis inhibition. This process suppresses internode elongation and reduces energy consumption until waterlogging stress is alleviated. In rice, the *SUB1A* locus is crucial for controlling this quiescence response by inducing fermentative metabolism to cope with waterlogging.

#### Escape response

In contrast, the *SK1* and *SK2* loci stimulate internode elongation under flood stress by promoting gibberellin biosynthesis in deepwater rice, allowing the plant to grow upward toward the water surface for air exchange. This mechanism enhances tolerance to waterlogging stress. Additionally, *ERFVII* transcription factors transduce extracellular signals into intracellular responses, inducing adaptive mechanisms such as glycolysis acceleration, stem elongation, aerenchyma formation, and increased oxygen transport.

**Figure 3:**
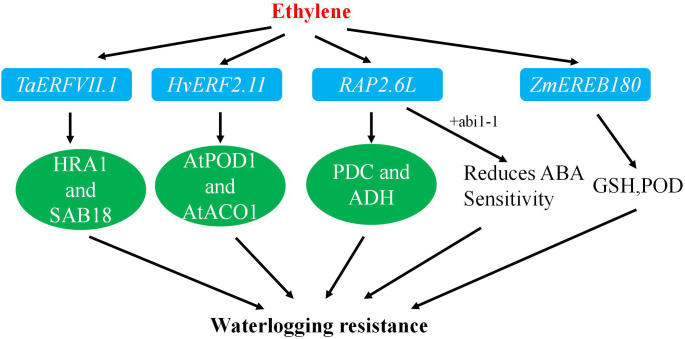
The pathway for improving waterlogging resistance.

Transgenic studies have shown that heterotopic expression of *ZmEREB180* in *Arabidopsis thaliana* increased survival rates after waterlogging stress. In maize, overexpression of *ZmEREB180* enhanced survival rates after prolonged waterlogging by promoting the formation of adventitious roots and regulating antioxidant levels (**Figure 3**) (**Table 2**).

Overexpression of *HvERF2.11* in *Arabidopsis* significantly improved tolerance to waterlogging. Transgenic plants exhibited a rapid increase in the expression of the antioxidant enzyme gene *AtPOD1* and higher activities of antioxidant enzymes and ADH compared to non-transgenic counterparts. These findings indicate that *HvERF2.11* plays a vital role in enhancing tolerance to waterlogging through the regulation of relevant genes and the enhancement of antioxidant enzyme activities.

Further studies have revealed that *RAP2.6L* transcripts are induced by waterlogging or ABA treatment. High expression of *RAP2.6L* reduced water loss and membrane leakage under waterlogging stress, with an associated increase in antioxidant enzyme gene expression and decreases in ABA sensitivity, suggesting its role in delaying submergence-induced premature aging through the ABI1-mediated ABA signaling pathway.

Moreover, the addition of MYC peptide-labeled *TaERFVII* to the N-terminus increased the survival rate of wheat and elevated the expression of waterlogging response genes. Stable expression of *TaERFVII.1* also enhanced the transcript levels of key genes in *Arabidopsis* and rice, indicating its significant role in wheat tolerance to waterlogging and its potential as a candidate gene for improving crop resilience to flooding.

The expression of *CgACO* in the waterlogging-tolerant variety (*Chrysanthemum zawadskii*) increased rapidly, reaching its peak 12 h after treatment and maintaining a high level for an additional 24 h. The high expression of *CgACO* may contribute to the accumulation of ethylene in *C. zawadskii*, supporting its tolerance to waterlogging stress (Zhang et al., 2018).

### Changes in root structure under waterlogging conditions

Under hypoxic conditions, *Zea nicaraguensis* can form a “tight” barrier for ROL at the base of the unstable root, a feature that is only weakly developed in the inbred line Mi29. The formation of this ROL barrier enhances plant resistance to waterlogging. Crossbreeding *Hordeum marinum*, which possesses water resistance and forms a ROL barrier, with wheat, which has water resistance but lacks the ROL barrier, results in a bifurcating effect that expresses some ROL barrier functionality and waterlogging resistance from its parents (Malik et al., 2011). Similarly, introducing the ROL barrier and stomatal characteristics of *Z*. *nicaraguensis* into maize could be a promising strategy for improving waterlogging resistance in maize (Abiko et al., 2012).

Anoxic preconditioning has been shown to enhance maize tolerance to waterlogged conditions. Tolerance to excess soil moisture (ESM) primarily relies on stress avoidance mechanisms through anaerobic metabolism to circumvent hypoxia (Zaidi et al., 2003). Hz32 and Mo17, the expression of 32 water-responsive miRNAs, were downregulated in the soaking coronal roots of maize seedlings. Several known miRNA targets involved in transcriptional regulation and ROS elimination were identified, shedding light on how miRNAs contribute to the morphological adaptation of maize under waterlogging stress (Zhai et al., 2013).

Understanding the genetic basis for the differences in response to waterlogging stress among maize inbred lines can provide new gene loci for enhancing tolerance to waterlogging through molecular marker-assisted breeding (Zhang et al., 2013).

Understanding the genetic basis for the differences in response to waterlogging stress among inbred maize lines can provide new gene loci for enhancing tolerance to waterlogging through molecular marker-assisted breeding. Genetic regulation of the polyamine pathway represents a potential method for improving plant tolerance to abiotic stress. Moreover, the mechanisms by which polyamines participate in plant abiotic stress tolerance have been explored, with an emphasis on the interplay between polyamines, abscisic acid, and nitric oxide in plant stress responses. Research has also focused on the interaction of polyamines with reactive oxygen species, ion channels, amino acids, and carbon metabolism (Shaw and Meyer, 2015). The water-tolerant allele *qWT_Gm03* exhibits strong root plasticity under waterlogged conditions, promoting root growth in non-stressed situations and potentially enhancing tolerance to waterlogging through improved water and nutrient absorption (Ye et al., 2018).

## Conclusion and prospect

Waterlogging stress is one of the most significant abiotic stresses impacting plant growth and agricultural productivity. Under waterlogged conditions, plants exhibit a range of complex adaptive mechanisms, including morphological adjustments, physiological and metabolic changes, and gene expression regulation. These adaptations involve not only individual genes or pathways but also a sophisticated network operating at multiple levels. Current research on crop tolerance to waterlogging primarily focuses on aspects such as morphology, structural changes, physiological biochemistry, metabolic pathways, and gene signaling regulation.

The most effective strategies to enhance plant tolerance to waterlogging include 1) alleviating the impact of waterlogging through drainage and fertilization, 2) mitigating the effects of waterlogging by applying exogenous hormones, and 3) modifying the expression of relevant genes to combat waterlogging-related diseases.

Building on existing research, future studies on plant tolerance to waterlogging should concentrate on the following areas: 1) since waterlogging stress often coincides with other environmental stresses, such as drought, salinity, and temperature fluctuations, it is essential to investigate how plants coordinate their responses to these combined stresses. This understanding is crucial for breeding crops with comprehensive stress resistance. 2) Although several hormones and genes related to waterlogging have been identified, translating these findings into practical crop improvement strategies remains challenging. Future research should further elucidate the functions of these waterlogging-related genes and their mechanisms of action under varying environmental conditions. 3) Developing more waterlogging-tolerant genetic resources and utilizing segregating and natural populations to identify waterlogging-related genes is equally important for breeding waterlogging-resistant crops.

We believe that as research on this topic deepens and expands, a more comprehensive and clearer regulatory network for plant responses to waterlogging will emerge, providing valuable theoretical guidance for understanding plant–environment interactions and breeding waterlogging-tolerant crops.
